# Assessment of Blend PVDF Membranes, and the Effect of Polymer Concentration and Blend Composition

**DOI:** 10.3390/membranes8010013

**Published:** 2018-03-05

**Authors:** Imtiaz Ali, Omar A. Bamaga, Lassaad Gzara, M. Bassyouni, M. H. Abdel-Aziz, M. F. Soliman, Enrico Drioli, Mohammed Albeirutty

**Affiliations:** 1 Department of Chemical and Materials Engineering, King Abdulaziz University, Rabigh 21911, Saudi Arabia; imtiaz_che@hotmail.com (I.A.); mbassyoni@kau.edu.sa (M.B.); 2 Center of Excellence in Desalination Technology, King Abdulaziz University, P.O. Box 80200, Jeddah 21589, Saudi Arabia; obamaga@kau.edu.sa (O.A.B.); lgzara@kau.edu.sa (L.G.); e.drioli@itm.cnr.it (E.D.); mbeirutty@kau.edu.sa (M.A.); 3 Department of Chemical Engineering, Faculty of Engineering, Port Said University, Port Said 42511, Egypt; 4 Chemical Engineering Department, Faculty of Engineering, Alexandria University, Alexandria 21544, Egypt; 5 Department of Civil Engineering, King Abdulaziz University, Rabigh 21911, Saudi Arabia; mfsoliman@kau.edu.sa; 6 Civil Engineering Department, Aswan University, Aswan 81528, Egypt; 7 Institute on Membrane Technology, ITM-CNR, c/o University of Calabria, Via P. Bucci, cubo 17/C, 87036 Rende, CS, Italy; 8 Mechanical Engineering Department, King Abdulaziz University, Jeddah 21589, Saudi Arabia

**Keywords:** PVDF membrane, blended membranes, mixed–solvent, membrane distillation

## Abstract

In this work, PVDF homopolymer was blended with PVDF-co-HFP copolymer and studied in terms of morphology, porosity, pore size, hydrophobicity, permeability, and mechanical properties. Different solvents, namely N-Methyl-2 pyrrolidone (NMP), Tetrahydrofuran (THF), and Dimethylformamide (DMF) solvents, were used to fabricate blended PVDF flat sheet membranes without the introduction of any pore forming agent, through a non-solvent induced phase separation (NIPS) technique. Furthermore, the performance of the fabricated membranes was investigated for pressure and thermal driven applications. The porosity of the membranes was slightly increased with the increase in the overall content of PVDF and by the inclusion of PVDF copolymer. Total PVDF content, copolymer content, and mixed-solvent have a positive effect on mechanical properties. The addition of copolymer increased the hydrophobicity when the total PVDF content was 20%. At 25% and with the inclusion of mixed-solvent, the hydrophobicity was adversely affected. The permeability of the membranes increased with the increase in the overall content of PVDF. Mixed-solvents significantly improved permeability.

## 1. Introduction

Water treatment can be carried out by different techniques such as: (i) Mechanical; (ii) Biological; and (iii) Physical /chemical processes, i.e., anodic oxidation [1,2,3]. Membrane separation is a physical chemical treatment process. Mass separating agents (MSA) are getting much attention in chemical technology and are being used in a wide variety of applications in almost all industrial sectors. Over the last few decades, membrane technology has become an important separation method and a suitable means of dealing with problems such as water scarcities, protection of the environment, and energy consumption. The useful property of a membrane is its ability to control the permeation rate of a desired component of a mixture while restricting other components. Various membrane applications require the different morphological structures of membranes ranging from a more porous structure for microfiltration (MF) to a more dense structure for reverse osmosis (RO) and a complete defect-free structure for gas separation (GS) and pervaporation (PV). On the basis of materials, membranes can be grouped as organic or inorganic. Organic membranes are made from cellulose acetate (CA), polyacrylonitrile (PAN), polysulfone (PSF), polyethylene (PE), polypropylene (PP), polyvinylidene fluoride (PVDF), polytetrafluoroethylene (PTFE), and polyamides (PA).

By virtue of its excellent amalgamation of properties and processability, poly(vinylidene fluoride) (PVDF) is available in a wide variety of molecular weights to fulfill typical manufacturing requirements. Thanks to its remarkable physicochemical properties, such as inertia to various solvents, acids, and oils, it has found a broad employment in process industries such as separation using membranes, including membrane distillation (MD), ultrafiltration (UF)/microfiltration (MF), and pervaporation (PV), etc. [4,5,6].

Generally, polymeric membranes used for micro- and ultrafiltration are prepared by phase inversion via immersion precipitation, which has been widely adopted for the preparation of asymmetric membranes [6,7,8,9]. In this method, a polymer solution is cast in a thin layer and immersed in a coagulation bath which contains a non-solvent. When the dope solution is immersed, a liquid-liquid demixing or precipitation phenomenon takes place and leads to the formation of the porous solid film. The structures of the developed membranes depend on the composition of the dope solution and coagulation bath [10,11,12]. PVDF is a semi-crystalline polymer which displays exceptional thermal, chemical, and oxidation resistances vis-a-vis corrosive chemicals such as acids, bases, oxidants, and halogens. The crystalline phase of the polymer offers thermal stability, whereas the amorphous phase provides the required membrane flexibility and good mechanical and film-forming properties [13,14,15,16]. In terms of hydrophobicity, although PVDF lacks PP and PTFE, the selection of suitable solvents for PVDF is facile. This is the main reason why PVDF is also still the best choice as a membrane material for MD and membrane contactor applications [17].

In recent years, a great deal of work has been dedicated to enhancing the strategy of materials selection to improve its performance [18].

In recent years, a great deal of work has been dedicated to enhancing the performance of PVDF membranes for MD by optimizing the morphological structure, increasing the hydrophobicity, improving the surface against fouling tendency, improving the mechanical strength, and enhancing the chemical resistance [18,19,20]. In this regard, the role of PVDF dope solution composition [21], solvents [22], and fabrication process parameters [23,24] has been considered. Besides these, the use of additives [25,26], novel PVDF copolymers [3], mixed solvents [27], and composite structures [17,28,29] is also getting much attention.

For a given polymer, e.g., PVDF, the average molecular weight of the polymer determines the interactions between the molecules of the polymer of different sizes and polymer mass, polymer mass and solvent, polymer mass and pore forming agents, and overall dope solution properties such as viscosity, solubility, and crystallinity, which are important parameters governing the phase separation process and mass transfer characteristics of the fabricated membrane. It is thus reasonable to believe that polymer molecular weights may have profound impacts on membrane properties and performance. But still, high-performance PVDF membrane fabrication is challenging and very hard to achieve [30]. The objective of this study is to assess the effect of a blending ratio of a low molecular weight PVDF copolymer with a high molecular weight PVDF homopolymer on physicochemical characteristics and permeability performance of produced membranes. A special point of focus is to explore the role of blending PVDF copolymer, polymeric concentration in dope solution, the choice of solvent or mixed-solvents, and membrane fabrication protocol during NIPS.

## 2. Experimental Work 

### 2.1. Materials

Solef^®^ 6020 (ultra high molecular weight PVDF homopolymer, M. wt 670–700 kDa) and Solef^®^ 21015 (medium molecular weight PVDF-co-HFP copolymer, M. wt 290–310 kDa) commercial polymers were provided by Solvay Specialty Polymers Italy S.p.A. *N*-Methyl-2 pyrrolidone (NMP), Tetrahydrofuran (THF), and Dimethylformamide (DMF) solvents were purchased from Sigma Aldrich, Germany. Iso-propanol and Glycerin were acquired from Sigma Aldrich, Germany.

### 2.2. Membranes Preparations 

Membranes were fabricated by the NIPS technique. The main hindrance in this technique is the lack of a predictable and methodical process in solvent system selection. Solvent plays a crucial role in determining the final membrane structure, properties, and performance. Higher polymer chain mobility is mandatory during casting, which is directly affected by both polymer-polymer and polymer-solvent interactions. If the dissolution of polymer molecules in a solvent is easy, then a consistent polymer distribution is achieved. For this study, three solvents with different boiling points were selected (NMP—B. Pt. 202 °C, DMF—B. Pt. 153 °C, and THF—B. Pt. 65 °C).

PVDF dope solutions were prepared on stirring hotplates and were kept at 100 °C for 24 h. The solutions PR-01, PR-02, and PR-03 were prepared with a 20% PVDF polymer concentration and PR-04, PR-05, and PR-06 with a 25% PVDF polymer concentration were prepared in NMP solvent, whereas the rest contained 25% of PVDF polymers dissolved in a mixture of solvents. The details of polymer dope solution compositions are described in Table 1.

**Figure 1:**
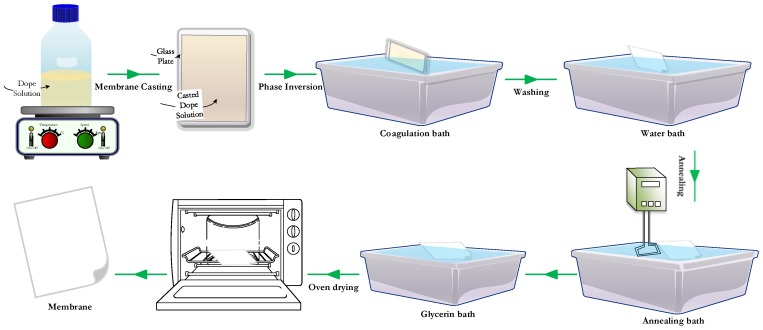
Schematic diagram of flat sheet membrane fabrication process through NIPS.

Membranes were cast using an automatic casting machine (Erichsen Unicoater 409, Erichsen: Hemer, Germany) on a glass plate (20 cm × 30 cm) with a fixed knife thickness of 400 µm. After casting, the glass plate containing thin polymeric film was first exposed to air for 90 s and then subjected to phase inversion in a coagulation bath for 10 min. The bath was prepared from 30% isopropanol (non-solvent) in water and maintained at 10 °C. During the phase inversion, thin polymeric films were separated from the glass. Membranes were annealed at 70 °C for 30 min inside a hot water bath. After annealing, the membranes were placed in a glycerin and non-solvent (water) bath for a period of approximately 30 min. Glycerin fills the pores of the membrane and prevents them from collapsing during storage prior to use in a filtering device. Membranes were then dried in an oven for 3 h at 60 °C to get rid of the remaining non-solvent. Prior to the testing, the membrane samples were just flushed with distilled water for glycerin removal. The process is described schematically in Figure 1 and the conditions are summarized in Table 2.

### 2.3. Membrane Characterization

Field Emission Scanning Electron Microscopy (FEI, Quanta FEG 450, Thermo Fisher Scientific: Census Bureau, OR, USA) was used to characterize the morphologies of the PVDF membranes. Membrane samples were attached to the grid using carbon copper tape and sputtered with gold by means of a sputter coater (Quorum Q150R ES, Quorum Technologies Ltd.: Ashford, Kent, UK). The voltage was set at 10 kV for cross section and surface images. Membranes were fractured using liquid nitrogen to obtain the cross-section images. The membrane surface morphology and roughness were characterized by atomic force microscopy (AFM) using the AFM XE-7-Park system at contact mode by placing AFM at the Vibration Isolation System inside the Acoustic Enclosure at room temperature.

Contact angle measurement is the most convenient way to characterize the wetting ability of a membrane surface. It is determined as the angle between the solid surface and a tangent to the curved surface of the drop at three phase contact. The contact angle is inversely related to the wetting ability of a solid surface by a liquid. Beside surface charge, such measurements are also affected by capillary forces within pores, roughness, and heterogeneity [31].

**Figure 2:**
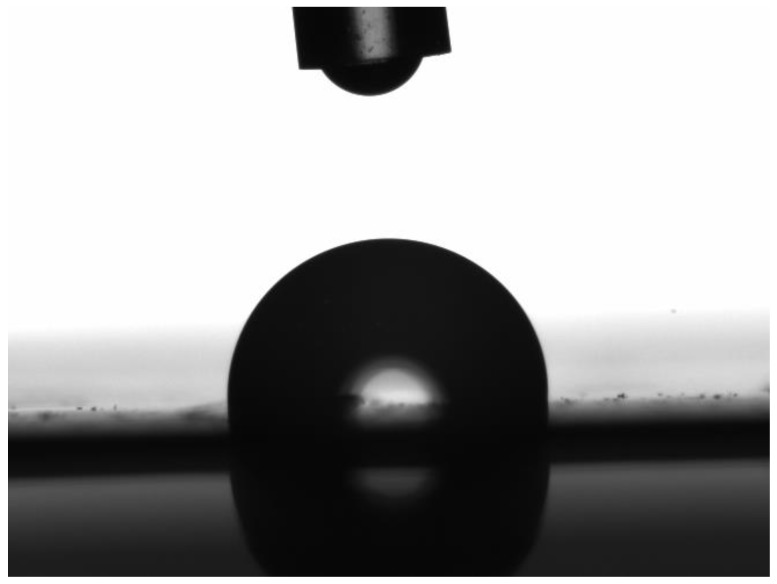
Captured image from dynamic contact angle measurement4.

An Attension Theta tensiometer was used to measure the contact angle of the prepared membranes using the Sessile drop method. Through a very fine capillary, 4 μL of a de-ionized water droplet was applied to the membrane surface (see Figure 2) and the contact angle was determined dynamically by One Attension image analysis software.

A capillary flow porometer 3 G-hz (Quantachrome Instruments: Delray Beach, FL, USA) was used for determining membranes’ bubble point and pore size distribution, as described elsewhere [20]. For each membrane type, different samples were fully wetted using low surface tension inert fluid (Porofil, 16 dyne/cm). Three measurements of different locations were averaged to attain the pore size distribution for each membrane sample.

The porosity of membranes was measured by the gravimetric method reported in the literature [32]. According to this method, the average membrane porosity is determined as the overall void fraction, calculated as the volume of the pores divided by the total volume of the membrane. Perfectly dried membrane samples were weighed with a precision balance. Samples were then immersed in kerosene for 24 h and weighed again. The overall porosity *ε_m_* was determined using the following formula:

(1) $$\varepsilon_{m}\left(\%\right)=\frac{\left(\frac{w_{1}\;-w_{2}}{\rho_{k}}\right)}{\left(\frac{w_{1}\;-w_{2}}{\rho_{k}}\right)+\frac{w_{2}}{\rho_{pol}}}\times 100$$

where *w*_1_, *w*_2_, *ρ_k_*, and *ρ_pol_* are the mass of the wet membrane, the mass of the dry membrane, the density of kerosene oil (0.82 g/cm^3^), and the density of polymer (PVDF = 1.78 g/cm^3^), respectively.

For each membrane type, three measurements were taken. Average values and the standard deviation were calculated.

### 2.4. Membrane Pure Water Permeability

#### 2.4.1. Pressure Driven 

Permeability is basically the ability of certain substances to pass through a barrier. Membranes play many different functions when it comes to permeating some elements while retaining others. The pure water permeability test of the elaborated membranes was carried out using a cross flow disc holder, EPDM version 90 mm purchased from Sterlitech, holding the effective membrane area at 50 cm^2^. Before the test, membranes were wetted in ultra-pure water for 3 h. Membranes were initially compacted at a 5 bar transmembrane pressure (TMP) for one hour. Permeability measurements were conducted at ambient temperature and at constant cross-flow (1 L·min^−1^). The pressure was varied from 1 to 4 bar during the test.

The permeation flux is defined by the following equation:

(2) $$J_{w}=\frac{\Delta V}{A\times \Delta t}$$

where $J_{w}$ = pure water volumetric flux [L·m^−2^·h^−1^]

ΔV = permeate volume [L]

A = effective area of the membrane [m^2^]

Δt = time [h]

**Figure 3:**
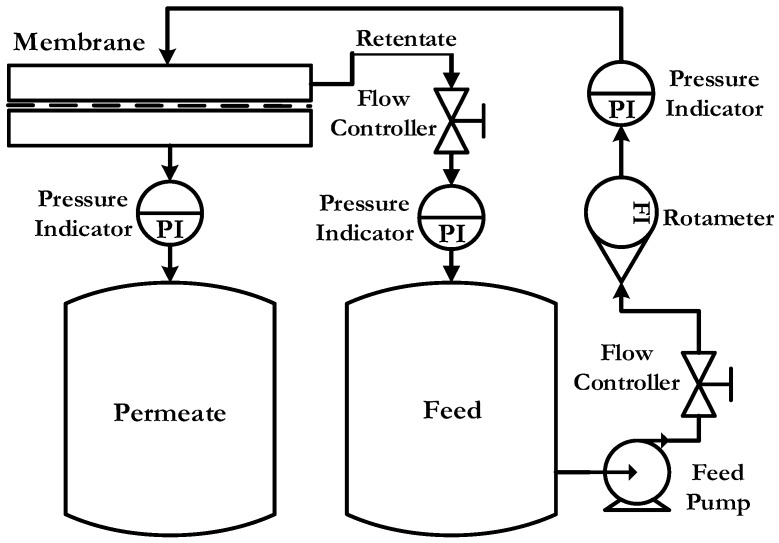
Process flow diagram of lab scale flat sheet membrane permeability test setup.

The experimental set-up is shown in Figure 3.

#### 2.4.2. Direct Contact Membrane Distillation

**Figure 4:**
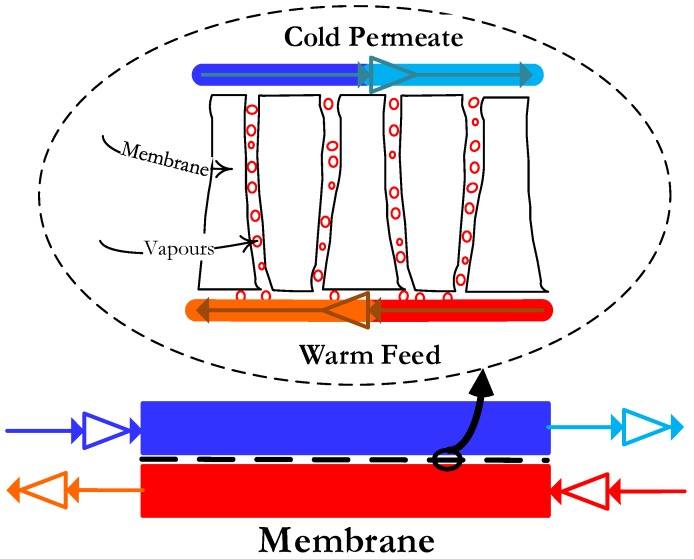
Schematic diagram of Direct Contact Membrane Distillation (DCMD) mechanism.

In DCMD, warm feed solution is maintained directly in contact with the cold permeate on the other side of the hydrophobic membrane. The temperature difference across the membrane causes the vapor pressure to change. As a consequence, volatile molecules turn into vapors at the hot liquid-vapor interface, cross the membrane through the pores, and condense in the cold vapor-liquid interface, as illustrated in Figure 4.

**Figure 5:**
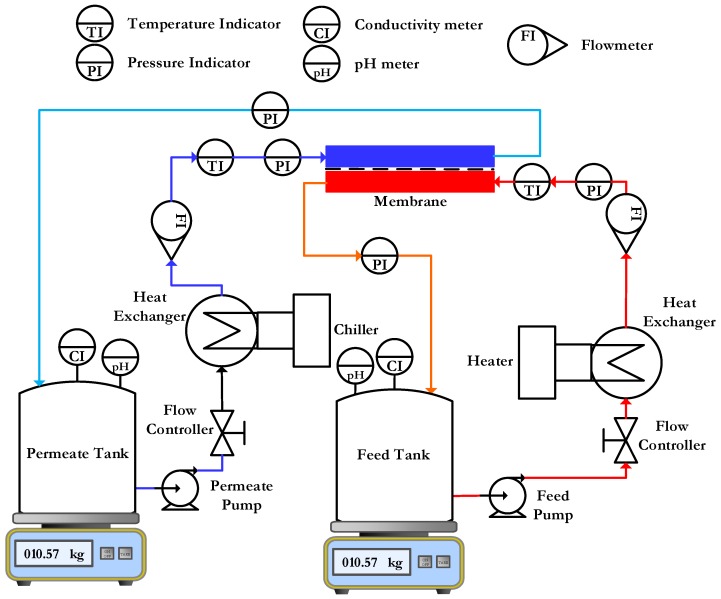
Process flow diagram of the lab scale flat sheet DCMD experimental setup.

The process flow diagram of the lab-scale flat sheet DCMD experimental set-up is shown in Figure 5. It is a custom set-up made from DeltaE S.r.l. There are two independent feed and permeate cycles in this configuration. One loop is for the hot feed and other is for the cold permeate. Both loops contain similar process components and controls.

The fabricated membranes were tested for pure water flux using the DCMD process to evaluate their performances as MD membranes under similar test conditions, as given in Table 2.

The permeate mass flux ($J_{p}$) through the membrane was calculated from Equation (3):

(3) $$J_{p}=\frac{\Delta m}{A\times \Delta t}$$

where Δm is the permeate mass, A is the effective area of membrane, and Δt is the operating time.

## 3. Results and Discussion

Membranes may vary significantly in their structure and eventually in their functionalities. To identify the utility of a membrane in a certain separation process, it is mandatory to characterize its structure and mass transport properties.

### 3.1. Mechanical Properties

The thickness of a membrane is an important parameter as it gives information on both the mechanical strength and the fluxes to be expected through a membrane. The decrease in membrane thickness increases the permeate flux but at the cost of higher heat loss and mechanical properties. It is reported that for MD, 30–60 μm thick membranes are ideal [33].

Membrane thickness was measured at four different points on every membrane with the help of a micrometer and is reported as an average. Strength properties were determined on the universal testing machine INSTRON 8502: Norwood, MA, USA, Servo-hydraulic type. Each sample was stretched uni-directionally at a constant rate of 5 mm/min; the gauge length was 50 mm.

The mechanical properties of the blended membranes in terms of the thickness, tensile stress at maximum load, tensile stress at break, modulus, and elongation at break are listed in Table 3.

Ultimate tensile strength (UTS), fracture strength (FS), and elongation at break were increased with the increase in overall PVDF content in the dope solution. The dependency of the mechanical properties of the membrane on the polymer molecular weight and the polymer concentration in dope solution has been well established, where the increase in any of the two parameters improves the mechanical properties of the membrane. In general, the data reported in Table 3 confirms this relationship. For the membrane prepared with NMP solvent alone (PR-01 through PR-06), the average UTS increased from 2.81 to 4.98 MPa as the PVDF polymer concentration increased from 20 to 25 wt %. The fracture strength and elongation at break have similar trends as well. However, Young’s modulus (E) decreased with the increase in overall PVDF content, which indicates that the membranes became flexible and less stiff. The unexpected decrease of Young’s modulus can be explained by the increase in percentage of low molecular weight polymer in the dope solution. The blending of a low molecular weight polymer with a high molecular weight polymer affects the viscosity of the dope solution and subsequently all other properties of the membrane. The higher the percentage of low molecular weight polymer in the polymer blend, the lower the values of the mechanical properties of the membrane at the given total polymer concentration. The only exception to this trend was PR-01, whereas due to the low percentage of copolymer, the viscosity of the obtained dope solution was higher. With an increase in the viscosity of dope solution, the miscibility of the solution is reduced and therefore the solution thermodynamic stability is enhanced, which resulted in a low porosity value and a wider bottom layer of spongy structure However, the mechanical properties of the membranes prepared with mixed-solvents were relatively higher compared to the ones prepared with NMP as the solvent; the only exception was PR-10. This means the membranes made from mixed-solvent became stiffer as well as tougher. The results reported in this work exhibited superior mechanical properties compared to the previous published study [34,35].

### 3.2. Membrane Structure

**Figure 6:**
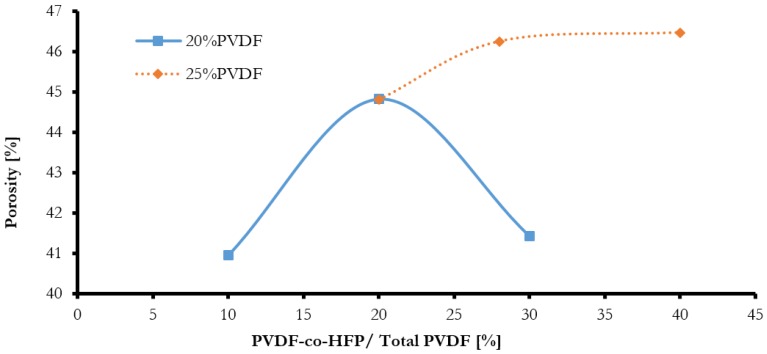
Effect of PVDF-co-HFP quantity on the blended membrane porosity with NMP as the solvent.

Membrane porosity and membrane pore size distribution results are displayed in Table 4. The porosity of the elaborated membranes increased slightly from 41 to 46% when the overall concentration of PVDF was increased from 20 to 25% with NMP as the solvent, as shown in Figure 6. Similarly, the porosity also increased when the concentration of PVDF-co-HFP copolymer was increased in the polymer blend, with the exception of PR-03. It is known that for a single polymer casting solution system, the viscosity of the solution increases and hence the porosity of casted membrane decreases with the increase in polymer molecular weight or the polymer concentration. However, when the casting solution is prepared from a blend of polymers of different molecular weights, the viscosity will depend not only on the total concentration, but also on the ratio of different components of the system. The reported increase in porosity with the increase of PVDF concentration can be explained by the higher percentage of copolymer relative to the homopolymer. By comparing the data from Table 3 and Table 4, it can be seen that the variations of porosity and membrane thickness have a similar pattern. The membrane thickness increased from 135 to 150 μm when the polymer concentration was increased from 20 to 25% with NMP as the solvent. The role of the viscosity factor on membrane porosity and thickness can be seen from the SEM images of cross sections of membranes. As the viscosity decreases with the increase of copolymer percentage, the thickness of the finger-like top layer of the membrane increases, resulting in development of a membrane with a high porosity and large thickness.

**Figure 7:**
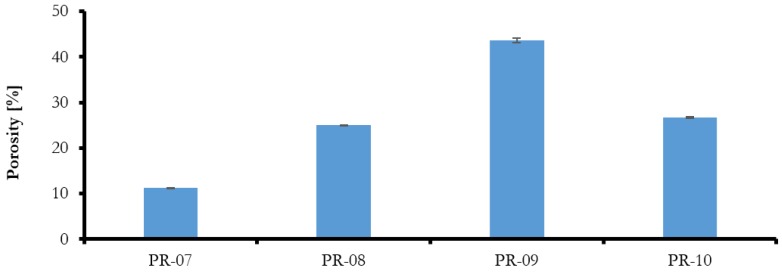
Effect of different solvent on blended membrane porosity.

The results of porosity, shown in Figure 7, with different solvents, indicate different values, but it can be seen that when NMP was used as a solvent, the porosity was increased. This could be due to the high diffusivity of NMP compared to THF and DMF in water during the phase inversion process. It can be seen that the best porosity was obtained when a mixture of NMP-THE-DMF was used (PR-09). The membrane prepared with NMP-THE-DMF mixture showed comparable porosity values to the membrane prepared with NMP solvent alone (PR-06). Therefore, it could be stated that the mixing of different solvents at the given ratios has no positive effects on porosity and pore size of the membrane.

In the literature, both high (around 85%) and low (around 25%) porosity values are reported for a blended PVDF membrane [36,37,38].

Membranes having a higher pore volume usually exhibit a higher performance in terms of the rate of evaporation [38]. From the previously reported results [32,36,39] and from this study, it can be assumed that the addition of copolymer, its amount, overall PVDF concentration in the solvent, and mixed-solvent in dope solution, affected porosity. Therefore, all these parameters must be selected carefully in order to get the desired PVDF membrane structure. The mean pore size of the fabricated membranes ranges from 0.02 to 0.08 μm (see Table 4), which is smaller than the PVDF membranes used in the MD studies elsewhere [40,41,42,43].

The relative hydrophobicity values of fabricated membranes in terms of dynamic contact angles are reported in Table 4. Results show that when the total concentration of the PVDF was 20% of the blended membranes and the amount of PVDF-co-HFP copolymer was increased, there was a slight increase in hydrophobic character. Conversely, when the total concentration of the polymer was 25% and the amount of PVDF-co-HFP copolymer was increased, there was a slight decrease in hydrophilicity.

**Figure 8:**
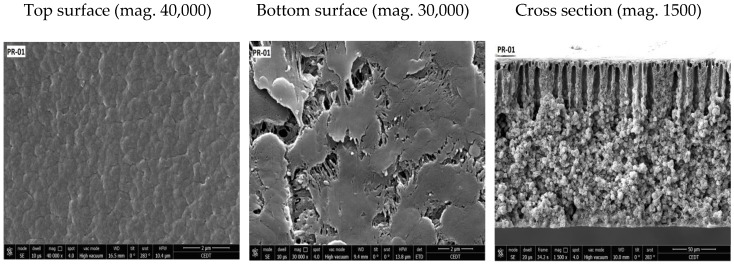

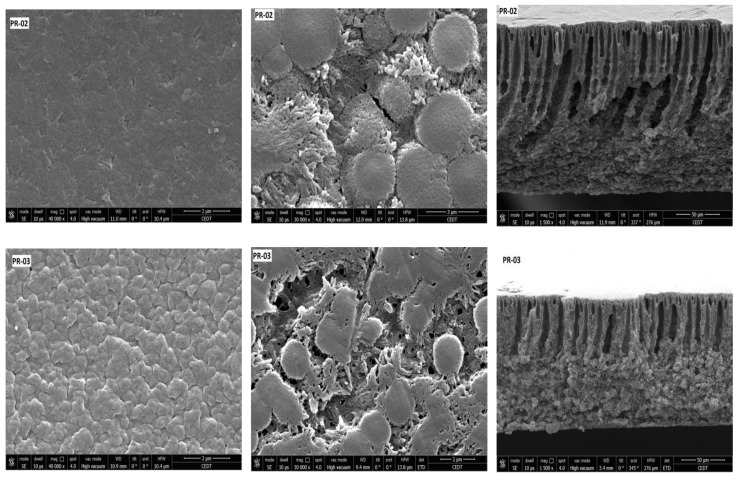
FESEM images of blended PVDF membranes films. Components of the dope solution: 20 wt % Solef^®^ 6020 PVDF homopolymer and Solef 90^®^ 21015 PVDF copolymer, 80 wt % NMP as a solvent. Percentage of the copolymer in the overall PVDF blend: PR-01: 10%, PR-02: 20%, PR-03: 30%.

**Figure 9:**
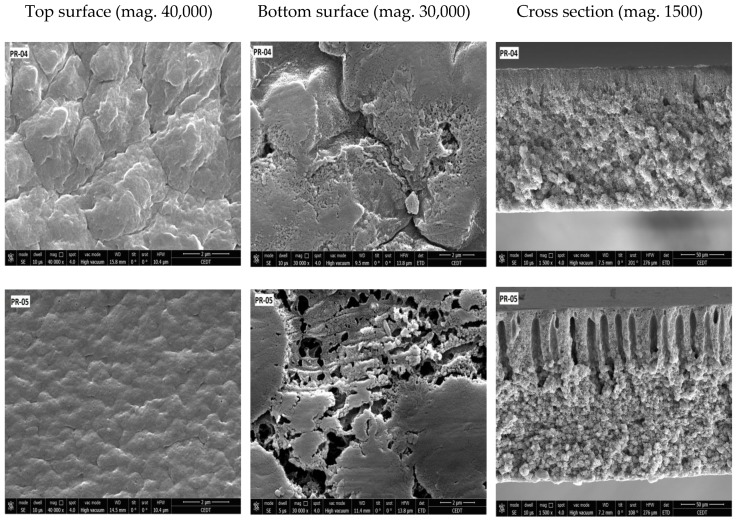

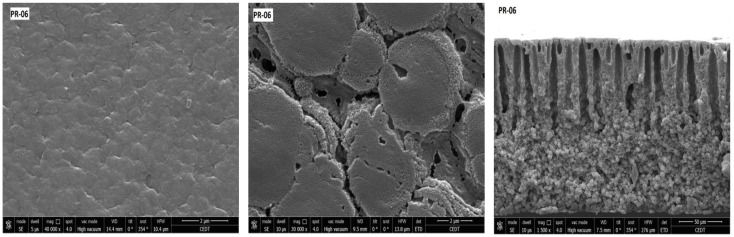
FESEM images of blended PVDF membranes films. Components of the dope solution: 25 wt % Solef^®^ 6020 PVDF homopolymer and Solef 90^®^ 21015 PVDF copolymer, 75 wt % NMP as a solvent. Percentage of the copolymer in the overall PVDF blend: PR-04: 20%, PR-05: 28%, PR-6: 40%.

Secondly, when the composition of the polymer was fixed and the solvents were changed with a different ratio, an overall decrease in hydrophobic character occurred, except for PR-09, which had a contact angle of 91.85°. The larger the contact angle of the membrane, the better it is for MD applications. It has also been reported that membranes having a contact angle between 90° and 105° are not really useful in most cases for MD applications [21,26]. When comparing the pattern of change of contact angle among membranes prepared with NMP as a solvent, it can be seen that the decrease in contact angle is associated with the increase in porosity and membrane surface indentations, as seen in Figure 8 and Figure 9. The contact angle as a measure of surface wettability is not only dependent on the intrinsic value of contact angle of the polymer material, but also depends on surface roughness, pore size, and heterogeneity of the surface. The effect of the roughness factor is clearer in the case of membranes prepared with solvent mixing where the contact angle was inversely related to roughness, for example, when the membrane top surface roughness increased from 3.15 nm (PR-09) to 4.28 (PR-07), the contact angle decreased from 91.850 to 83.460, respectively.

**Figure 10:**
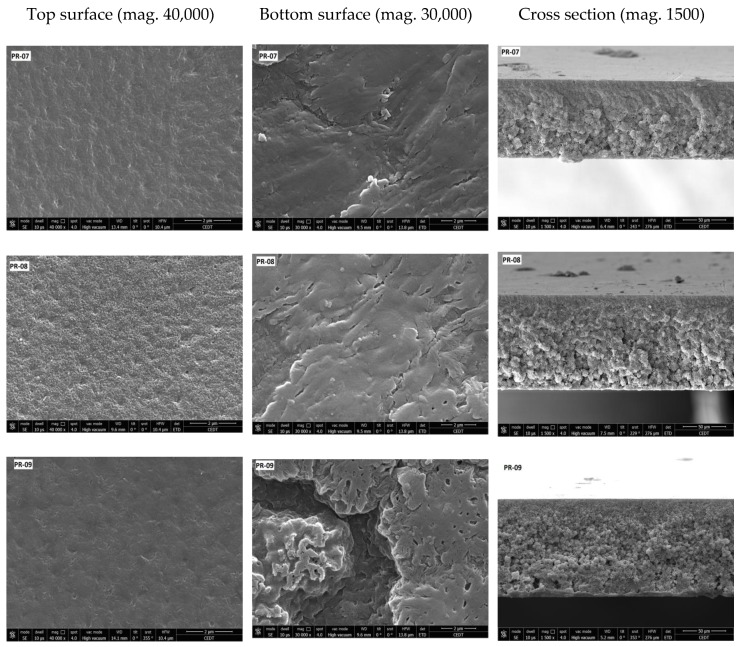

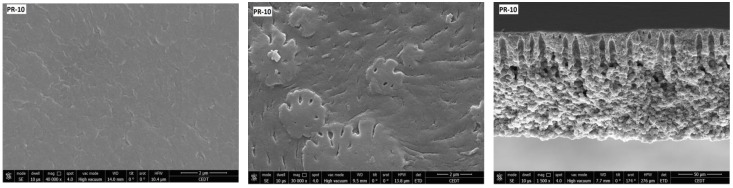
FESEM images of blended PVDF membranes films. Components of the dope solution: 15 wt % Solef^®^ 6020 PVDF homopolymer, 10 wt % Solef 90^®^ 21015 PVDF copolymer, 75% wt mixture of solvents, Percentage of solvents in dope solution: PR-07: THF-DMF (40–35%), PR-08: NPM-THF (40–35%), PR-9: NPM-THE-DMF (25–25–25%), PR-10: NPM-DMF (40–35%).

SEM images of the prepared membranes are shown in Figure 8, Figure 9 and Figure 10. All prepared membranes showed asymmetric structures in the cross-section SEM images. When NMP was used, the top layer of the membranes showed a finger-like structure, whereas the bottom layer was constituted of a spongy and globular structure. By increasing the amount of PVDF-co-HFP, the finger-like structure became deep. That is the reason why the porosity was increased with the addition of PVDF-co-HFP. On the other hand, when the mixture of solvent was used, the structure of the membrane became less asymmetric and spongy.

The top and bottom side of the membranes exhibited different structures. The top surface was less open and showed greater film integrity, whereas the bottom surface showed larger opening and was mostly composed of aggregated particles.

**Figure 11:**
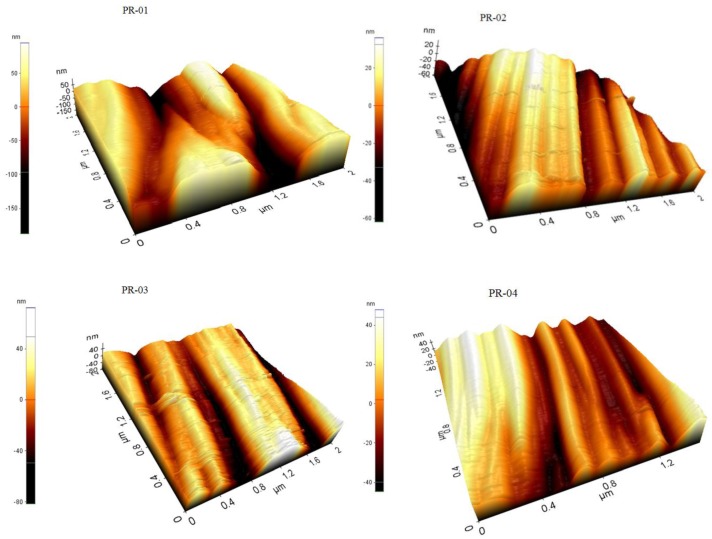

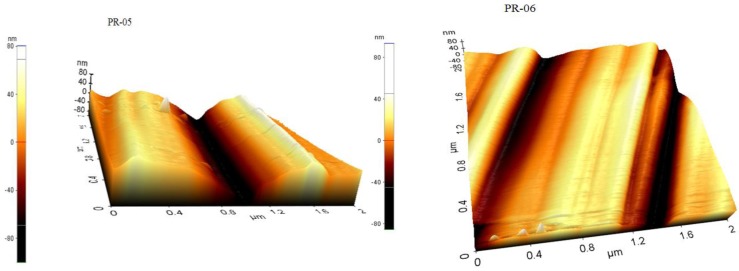
AFM images of blended PVDF membranes films prepared in NMP.

**Figure 12:**
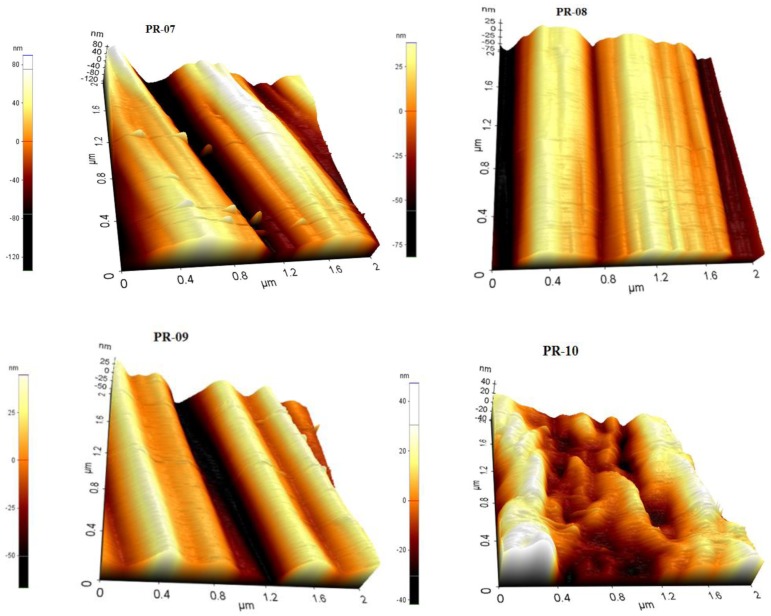
AFM images of blended PVDF membranes films prepared in solvents mixtures.

The surface roughness increases the possibility of bacterial deposition, which can affect the affinity between membrane surfaces and fouling [44]. Therefore, improving the surface smoothness of the membrane may reduce biofouling of the PVDF blended membranes. The surface morphology and roughness of the fabricated membranes were characterized by the AFM XE-7-Park system in contact mode. The AFM topographies of the PVDF blended membranes are shown in Figure 11 and Figure 12. The average value of roughness (Ra) for the top and bottom side are presented in Table 4. It is is evident from the lower roughness values of the top-side that the top surface is smoother than the bottom surface, which is in agreement with the SEM observations.

The result indicates that blending PVDF and solvent had a slight effect on the surface roughness. There is a decreasing trend of roughness at different concentrations and at different ratios of two PVDF polymers. The roughness of the skin layer of the top layer of the membranes slightly decreased with the addition of PVDF-co-HFP in the dope solution, which can be attributed to the reduction in the viscosity of the dope solution. This observation is in agreement with the previously reported study [45]. There is also decrease in roughness value when changing the different solvents. Chen et al. [46] reported the same behavior of roughness of the PVDF membranes using the different solvents and additives.

### 3.3. Membrane Pure Water Permeability

**Figure 13:**
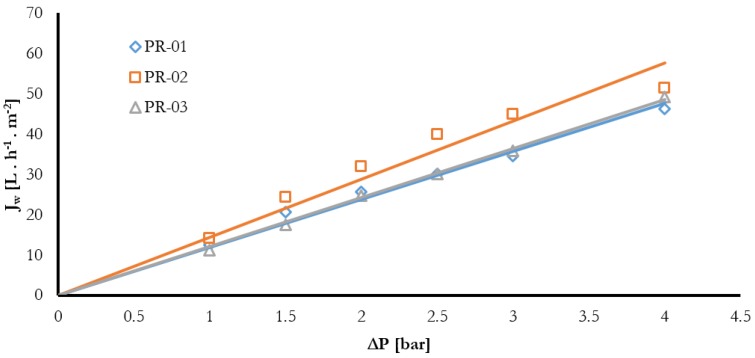
Pure water flux (Jw) as a function of trans-membrane pressure and PVDF-co-HFP amount (20% of total PVDF) with NMP as the solvent.

Figure 13 represents the evolution of pure water flux as a function of transmembrane pressure for PR-01, PR-02, and PR-03 membranes. The water permeation flux ($J_{w}$) varies linearly with the transmembrane pressure (ΔP) following Darcy’s law. ($J_{w}=\;L_{p}\;\Delta P$), where $L_{p}$ is the membrane hydraulic permeability. Similar trends were observed for the rest of the other membranes. The membrane permeability was determined by calculating the slope of the obtained curves and summarized in Table 5. It is shown that the permeability increased with the increase in overall composition of the PVDF from 20–25%. On the other hand, while changing the different solvents, the highest permeability was obtained for membrane PR-07, with a value of 35.31 L·h^−1^·m^−2^.

The permeate mass flux (*L_p_*) values through the membranes are listed in Table 5. The DCMD experiment for every membrane was replicated three times and the average flux value along with standard deviation is reported. These results demonstrate that the mass flux of permeate decreased with the increase in overall PVDF content. However, the obtained flux values were better when NMP was used as a solvent. It is clear from the table that with the increase of PVDF-co-HFP copolymer concentration, there was a slight decrease in permeate flux at 20% of the overall concentration of the polymers. On the other hand, the flux value increased with the increase of PVDF-co-HFP copolymer at 25% of the total polymer concentration when NMP was used as a solvent. The pure water permeate flux of PVDF membranes in the DCMD test is compared with the previously reported values in Table 6.

Feed temperature 70 °C, Permeate (condensate) temperature 20 °C, Feed flow rate 1.7 L·min^−1^, Permeate flow rate = 1.9 L·min^−1^.

The permeate flux value in the present study is lower than previously reported. This can be attributed to the smaller pore sizes of the prepared membranes due to kinetics (solvent evaporation and polymer precipitation).

**Figure 14:**
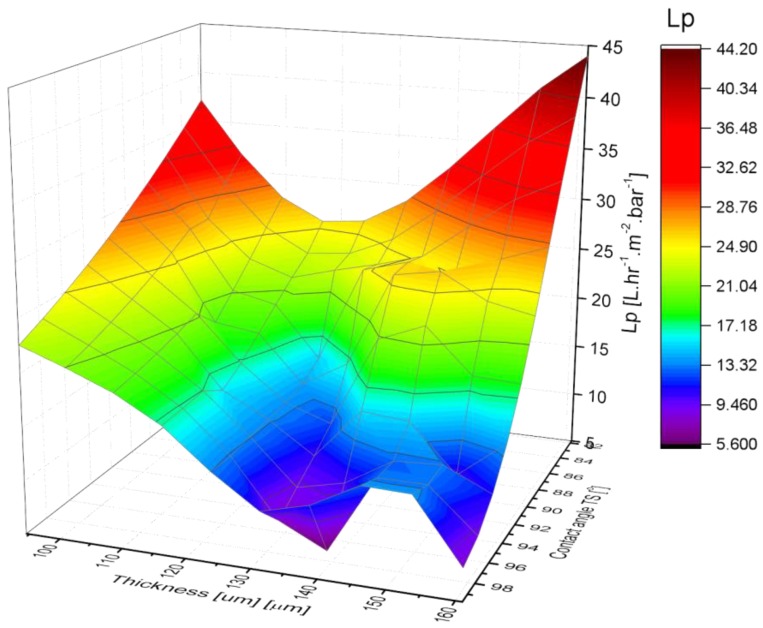
3D graph of PVDF membranes’ water permeability as a function of thickness and contact angle.

The variation of PVDF membranes water permeability as a function of contact angle and thickness is shown in Figure 14. The permeability of the PVDF membrane varies inversely to the thickness and contact angle during the pressure driven experiment.

**Figure 15:**
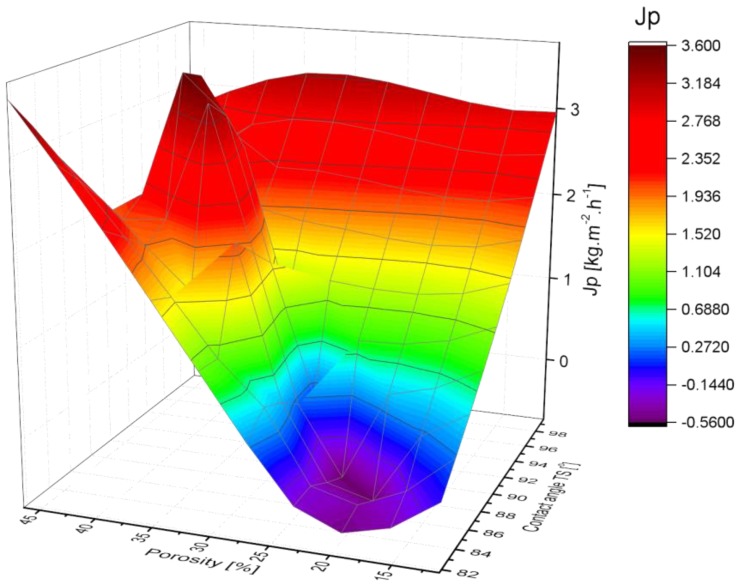
3D graph of permeate vapor flux as a function of porosity and contact angle.

The variation of PVDF membranes permeate flux as a function of contact angle and porosity is shown in Figure 15. The permeate flux of PVDF membrane varies directly to the porosity and contact angle during the DCMD experiment.

## 4. Conclusions

The series of blended PVDF membranes were fabricated by the NIPS method. The amount of polymers, blending ratio, solvent, mixed solvents, and other casting parameters affected the properties of the membrane. The main conclusions are as follows:

- Addition of PVDF-co-HFP copolymer, increasing the overall PVDF content, and use of mixed-solvent improved mechanical properties of the membranes.
- The porosity of the elaborated membranes increased with the increase in the overall content of PVDF and with the addition of PVDF-co-HFP copolymer.
- At 20% overall PVDF content, the addition of copolymer increased hydrophobicity and the opposite was true at 25% overall PVDF content. Mixed solvent had an adverse effect on hydrophobicity.
- The AFM results show that the prepared membranes have a relatively good smoothness.
- Permeability of the membranes increased with the increase in overall content of PVDF. Mixed-solvents significantly improved permeability.
- The results of the DCMD experiment demonstrate that the obtained membrane does not meet the required target to be used in the MD process without pore forming agent.

## Figures and Tables

**Table 1:** Composition of the casting solutions.

Membrane	PVDF Homopolymer	PVDF-co-HFP	NMP	THF	DMF
PR-01	18	2	80	-	-
PR-02	16	4	80	-	-
PR-03	14	6	80	-	-
PR-04	20	5	75	-	-
PR-05	18	7	75	-	-
PR-06	15	10	75	-	-
PR-07	15	10	-	40	35
PR-08	15	10	40	35	-
PR-09	15	10	40	-	35
PR-10	15	10	25	25	25

**Table 2:** Summary of condition for flat sheet membrane fabrication through NIPS.

Sr. No.	Parameter	Value
1.	Casting knife thickness	400 µm
2.	Casting knife speed	10 mm·s^−1^
3.	Time before immersion	90 s
4.	Coagulation bath	30% isopropanol in water
5.	Coagulation bath	180 s at 10 °C
6.	Washing bath (water)	15 min
7.	Annealing bath	30 min at 70 °C
8.	Glycerin bath	30 min
9.	Oven	3 h at 60 °C
10.	Indoor relative humidity	60%

**Table 3:** Mechanical properties of blended PVDF membranes.

Membrane	Thickness [μm]	Yield Strength [MPa]	Fracture Strength [MPa]	Young’s Modulus [MPa]	Elongation at Break [%]
PR-01	130.5 (±4.7)	2.56	3.01	50.77	26.86
PR-02	148.3 (±6.4)	1.78	1.82	76.42	9.11
PR-03	126.0 (±2.2)	2.08	2.72	66.03	17.21
PR-04	150.0 (±4.8)	4.01	4.05	58.19	29.32
PR-05	138.3 (±3.0)	3.17	3.94	50.88	34.77
PR-06	161.0 (±5.4)	4.01	4.41	25.25	51.33
PR-07	94.5 (±1.9)	5.22	7.37	60.33	55.32
PR-08	114.3 (±2.2)	5.53	9.62	82.26	62.36
PR-09	140.0 (±3.4)	6.13	10.56	87.12	69.44
PR-10	105.5 (±4.2)	3.59	4.22	45.52	36.21

**Table 4:** Morphological properties of membranes.

Membrane	Porosity [%]	Mean Flow Pore (MFP) Size	Contact Angle (Topside) [°] (±Std. Dev.)	Roughness (Topside) Ra [nm]	Roughness (Bottom Side) Ra [nm]
PR-01	40.95 (±0.70)	0.02	91.39 (±1.52)	3.49	39.49
PR-02	44.83 (±0.80)	-	99.70 (±1.04)	2.05	08.93
PR-03	41.43 (±0.27)	0.08	99.33 (±0.93)	3.28	14.34
PR-04	44.82 (±0.70)	0.05	96.33 (±0.86)	3.43	18.49
PR-05	46.25 (±0.00)	-	97.44 (±0.36)	3.13	13.74
PR-06	46.47 (±0.33)	0.02	90.11 (±0.13)	2.78	17.68
PR-07	11.22 (±0.05)	-	83.46 (±0.10)	4.28	33.13
PR-08	25.04 (±0.08)	-	81.71 (±0.50)	3.21	23.63
PR-09	43.63 (±0.50)	0.02	91.85 (±1.01)	3.15	18.42
PR-10	26.74 (±0.22)	0.02	88.33 (±1.45)	-	14.23

**Table 5:** Pure water permeability and permeate vapor flux of PVDF under the DCMD test using pure water as a feed and as a condensate.

Membrane	Water Permeability L_p_ [L·h^−1^·m^−2^·bar^−1^] (R^2^)	Permeate Vapor Flux [kg·m^−2^·h^−1^] (±Std. dev.)
PR-01	11.91 (0.977)	2.916 (±0.006)
PR-02	14.41 (0.923)	2.892 (±0.006)
PR-03	12.12 (0.998)	2.871 (±0.006)
PR-04	9.59 (0.994)	1.524 (±0.004)
PR-05	5.79 (0.997)	1.652 (±0.005)
PR-06	22.09 (0.982)	1.833 (±0.005)
PR-07	35.31 (0.976)	0.219 (±0.002)
PR-08	25.85 (0.969)	0.004 (±0.002)
PR-09	21.34 (0.997)	1.361 (±0.004)
PR-10	21.78 (0.977)	0.083 (±0.002)

**Table 6:** Pure water permeate flux of PVDF membranes in the DCMD process.

Mean Pore Size [μm]	Thickness [μm]	Feed Velocity [m·s^−1^]	T_f_ [°C]	Permeate Flux [kg·m^−2^·h^−1^]	Reference
0.22	126	0.1	40–70	3.6–16.2	[40]
0.22	120	0.145	36–66	5.4–36	[41]
0.22	110	0.23	40–70	7–33	[42]
0.2	100	1.2 L·min^−1^	30–46	7–41	[43]
0.02–0.08	95–161	1.7 L·min^−1^	70	0.004–2.9	Present study
